# Perioperative Dynamics of Tumor Cells into the Circulatory System During Vertebral Cement Augmentation in Metastatic Spinal Disease—A Prospective Pilot Study

**DOI:** 10.3390/jcm15218400

**Published:** 2026-10-28

**Authors:** Moritz C. Deml, Samuel F. Schaible, Carlo R. Largiadèr, Dominic Schärer, Lorin M. Benneker, Helena Mengolian

**Affiliations:** 1 Department of Orthopaedic Surgery and Traumatology, University Hospital Bern, Inselspital, University of Bern, 3010 Bern, Switzerland; samuel.schaible@insel.ch (S.F.S.);; 2 Department of Clinical Chemistry, University Hospital Bern, Inselspital, University of Bern, 3010 Bern, Switzerland; carlo.largiader@insel.ch (C.R.L.); dominic.schaerer@insel.ch (D.S.); 3 Sonnenhofspital Bern, University of Bern, 3006 Bern, Switzerland

**Keywords:** circulating tumor cells, spinal metastases, vertebroplasty, kyphoplasty, cement augmentation, tumor cell dissemination, AdnaTest, mRNA markers

## Abstract

**Background/Objectives**: Vertebroplasty (VP) and balloon kyphoplasty (KP) are well-established surgical interventions used to stabilize pathological fractures and severe spinal instability resulting from osteolytic spinal metastases. Both procedures increase intravertebral pressure, which may elevate the risk of hematogenous dissemination of tumor cells. However, data on circulating tumor cells (CTCs) in the perioperative setting remain limited. **Methods**: This prospective, single-center exploratory pilot study included 11 patients undergoing VP or KP for spinal metastases (breast cancer, *n* = 7; prostate cancer, *n* = 3; colon cancer, *n* = 1). Peripheral blood samples were collected preoperatively and at 1, 5, 10, and 20 min after cement injection and analyzed for CTC-associated, tumor-specific mRNA markers (AdnaTest^®^) using marker panels corresponding to each patient’s primary cancer type. Vertebral biopsies obtained prior to cement injection confirmed tumor infiltration of the treated levels. **Results**: In all patients with histopathologically confirmed tumor presence (9/10; 90%), one or more CTC-associated mRNA markers were detectable at one or more time points, most consistently at 5 and 10 min after cement injection, whereas the biopsy-negative patient showed no marker signal. In an exploratory analysis of the four patients with baseline GA733-2 positivity (three breast cancer and one colon cancer case), a 3.8-fold increase in marker levels was observed 5 min post-injection (nominal *p* = 0.026, 95% CI: 1.4–10.4; not adjusted for multiple comparisons); this effect was not sustained at 10 or 20 min. No significant correlation was found between cement volume, baseline marker levels, or vertebral location (thoracic vs. lumbar). **Conclusions**: In this pilot study, CTC-associated mRNA markers were detectable in peripheral blood during VP and KP, and marker positivity was concordant with the histopathological findings of the treated vertebra in this small cohort. The transient increase of a single marker in a small subgroup is hypothesis-generating and does not demonstrate the release of viable tumor cells. Whether perioperative strategies to limit tumor cell release are warranted needs to be evaluated in larger, controlled studies.

## 1. Introduction

Pathological fractures of the vertebral bodies due to spinal metastases represent an increasing clinical challenge [1]. Management strategies for these lesions—including both established fractures and high-risk, unstable vertebrae—range from conservative treatment and radiotherapy to surgical intervention [2]. Among surgical options, percutaneous cement augmentation using balloon kyphoplasty (KP) or vertebroplasty (VP) with polymethyl methacrylate (PMMA) has become widely accepted as standard practice [3]. These minimally invasive techniques are frequently employed in the treatment of spinal metastases, offering effective vertebral stabilization and pain relief with a relatively low complication rate [2,4].

In addition to known risks such as cement leakage, pulmonary embolism, and fat embolism [5,6], there is growing concern that KP and VP may facilitate hematogenous dissemination of tumor cells. The increased intravertebral pressure generated during cement injection may contribute to the release of circulating tumor cells (CTCs) into the peripheral bloodstream—a phenomenon that remains insufficiently understood in the perioperative context [7].

Several studies have reported increases in peripheral blood tumor cell counts during various surgical interventions, with intraoperative elevations in CTC levels associated with poorer clinical outcomes and reduced survival [8,9,10,11]. A review of the literature identified three case reports suggesting that KP may promote tumor cell dissemination from the vertebral body. Of these, two cases involved local tumor progression, and one described metastatic spread following the procedure [12,13]. The proposed underlying mechanism is a procedure-related rise in intravertebral pressure, which may force tumor cells into the circulatory system. This mechanism has previously been demonstrated in the context of osteoporotic vertebral fractures, where cement injection has been shown to displace intravertebral fat and blood into the venous system [14].

Mohme et al. conducted a preliminary investigation of CTC release during vertebral cement augmentation for osteolytic spinal metastases of various primary origins [7]. Peripheral blood samples were collected at three time points: preoperatively, 20 min after cement application, and 3–5 days postoperatively. CTCs were identified using the FDA-approved CellSearch^®^ system, which detects epithelial cell adhesion molecule (EpCAM)-positive cells. However, this assay primarily targets epithelial surface markers and may underestimate true tumor burden, particularly in advanced metastatic disease, where epithelial-to-mesenchymal transition can result in downregulation of EpCAM expression.

The objectives of this study were as follows: (1) to evaluate whether CTC-associated, tumor-specific mRNA markers are detectable in peripheral blood during VP and KP procedures using the AdnaTest^®^ platform, and (2) to describe the early perioperative dynamics of these markers in order to identify suitable blood sampling time points for future studies of tumor cell release during vertebral cement augmentation.

## 2. Materials and Methods

### 2.1. Study Design and Patients

Following approval by the local ethics committee, we conducted a prospective, single-center study involving 11 consecutive patients. Recruiting started after final approval by the local ethics committee was received. Inclusion criteria were: age ≥18 years, confirmed primary tumor type (breast, *n* = 7; prostate, *n* = 3; colon, *n* = 1), and thoracolumbar spinal metastasis resulting in either a confirmed or impending pathological vertebral body fracture. Additionally, all lesions had to meet a Spinal Instability Neoplastic Score (SINS) of ≥8. The choice between vertebroplasty (VP) and kyphoplasty (KP) was based on vertebral body integrity and kyphotic angulation. Vertebrae with a kyphotic angle >20° (between upper and lower endplates) and >80% endplate integrity were selected for KP.

### 2.2. Surgical Procedure

For KP, we used a 10-gauge transpedicular Jamshidi-type needle with a laterally open cement delivery system (Access Kit, DePuy Synthes, Dübendorf, Switzerland), followed by endplate reconstruction using a kyphoplasty balloon (Synflate^®^, DePuy Synthes). VP was performed using an 8-gauge transpedicular T-Lok bone marrow biopsy needle (Argon Medical, Plano, TX, USA). Prior to cement injection, histopathological specimens were obtained from the treated vertebral level to confirm tumor infiltration. In both procedures, polymethyl methacrylate (PMMA; Vertecem^®^, DePuy Synthes, Johnson & Johnson, New Brunswick, NJ, USA) was injected under continuous biplanar fluoroscopic guidance (lateral and anteroposterior) until the metastatic cavity was adequately filled between the endplates, or until cement leakage into the disc space or venous system was observed.

### 2.3. Blood Sampling and CTC Marker Analysis

Peripheral blood samples were collected in 8.5 mL ACD-A Vacutainer tubes (Becton Dickinson, Franklin Lakes, NJ, USA) via either a central venous catheter (*n* = 6; 54.5%) or a peripheral venous catheter (*n* = 5; 45.5%). Due to the study’s nature as a pilot study, the sampling route was not standardized by the study protocol; the vascular access placed by the anesthesia team for the individual patient was used, and the same access was used for all samples of a given patient. Sampling was performed preoperatively and at 1, 5, 10, and 20 min following cement application. All samples were processed within 36 h of collection.

Enrichment of CTCs was performed using AdnaTest^®^ CancerSelect Kits (Qiagen GmbH, Hilden, Germany), which are tumor-specific for breast, prostate, and colon cancer. For each time point, a 5 mL ACD-A blood sample was transferred to an Adna Tube, and cells expressing tumor-associated surface antigens were captured using immunomagnetic beads coated with the corresponding antibodies. Captured cells were separated using a magnetic particle concentrator, washed, lysed, and kept on ice for downstream analysis.

For molecular detection using the AdnaTest^®^ CancerDetect Kits, mRNA was extracted from the cell lysate with Oligo(dT)_25_-coated magnetic beads and reverse-transcribed into complementary DNA (cDNA). Tumor-specific markers and β-actin (as an internal control) were amplified by multiplex PCR. Amplicons were detected using the 2100 Bioanalyzer and DNA 1000 LabChip reagents (Agilent Technologies, Santa Clara, CA, USA). Marker panels were as follows: for breast cancer—GA733-2, MUC-1, and Her-2; for prostate cancer—PSMA, PSA, AR, and EGFR; and for colon cancer—GA733-2, CEA, and EGFR. Marker positivity was defined according to the manufacturer’s instructions: a sample was considered positive for a given marker if the corresponding PCR fragment was detected above the manufacturer’s peak-concentration threshold on the Bioanalyzer, provided that the β-actin fragment was present as internal control. Values below this threshold were classified as negative and were not replaced by imputed values; paired quantitative comparisons were performed in patients with a positive baseline value for the respective marker (Section 2.4). Samples were analyzed in the Department of Clinical Chemistry by personnel not involved in the surgical procedures; no formal blinding to sampling time point or histopathological result was implemented, and the analytical reproducibility of the assay was not formally assessed in this study. The assay detects tumor-associated mRNA transcripts in the enriched cell fraction; it does not enumerate intact cells or assess their viability. Throughout this manuscript, the terms “CTC marker” and “CTC-associated mRNA marker” therefore refer to the detection of such transcripts and not to the direct demonstration of viable circulating tumor cells.

### 2.4. Statistical Analysis

This was an exploratory pilot study: no primary marker and no primary time point were prespecified, and the sample size was determined by feasibility rather than by a formal power calculation. Quantitative expression levels of tumor-specific mRNA were normalized and compared with baseline at 5, 10, and 20 min using paired two-tailed t-tests in patients with a positive baseline value for the respective marker; changes are reported as fold changes with 95% confidence intervals. Given the small number of paired observations, the normality assumption could not be meaningfully tested, and no adjustment for multiple comparisons was applied; *p*-values are therefore reported as nominal, unadjusted values, and *p* < 0.05 was regarded as a signal warranting further investigation rather than as confirmatory evidence. Exploratory correlation analyses were performed to evaluate associations between marker levels and cement volume, vertebral level (thoracic vs. lumbar), or baseline marker positivity. Due to the limited sample size, multivariate regression analysis was not performed.

## 3. Results

### 3.1. Patient Characteristics

We included 11 patients with a mean age of 66.1 years (range: 39–77; SD: 11.5). Eight patients (73%) were female, and three (27%) were male. Ten patients (91%) underwent kyphoplasty (KP), and one patient (9%) underwent vertebroplasty (VP). The mean Spinal Instability Neoplastic Score (SINS) was 12.2 (range: 8–15; SD: 2.2). KP/VP was performed in the lumbar spine in 7 patients (64%) and in the thoracic spine in 4 patients (36%). Detailed demographics are presented in Table 1.

### 3.2. Detection of CTC-Associated mRNA Markers

One patient with prostate cancer was excluded from the marker analysis due to blood sample clotting, leaving a final evaluable cohort of 10 patients (breast cancer, *n* = 7; prostate cancer, *n* = 2; colon cancer, *n* = 1). Of the vertebral biopsies obtained, 9 of 10 (90%) were histopathologically positive for metastatic tumor cells. In the remaining patient (prostate cancer), metastatic involvement of the treated level had been established preoperatively by CT, together with an elevated serum prostate-specific antigen (PSA) level and multiple further osseous metastases; the intraoperative biopsy, however, showed no tumor cells, and no CTC-associated marker was detectable in this patient at any time point. Because a negative vertebral biopsy in a patient with metastatic disease may reflect a sampling limitation rather than the absence of tumor, this patient was not regarded as a true disease-negative control.

At least one CTC-associated mRNA marker was detectable in all 9 patients with histologically confirmed spinal metastases at both 5 and 10 min after cement application (100%). In 5 of these patients (56%), a marker signal was additionally detectable at 1 or 20 min post-procedure. These detection rates refer to binary marker positivity and not to quantitative changes in marker levels.

Among patients with breast cancer (*n* = 7), tumor-specific mRNA markers were identified as follows: MUC-1 in 6 patients (86%), GA733-2 in 5 (71%), and Her-2 in 3 (43%). In the single colon cancer case, GA733-2 was detected at all post-cementation time points, while EGFR and CEA remained below detection thresholds. In the single evaluable prostate cancer patient with histologically confirmed metastasis, one or more prostate-specific mRNA markers were detectable at 5 and 10 min after cement application; given the single case, no marker-specific analysis is presented for the prostate cancer group.

**Figure 1:**
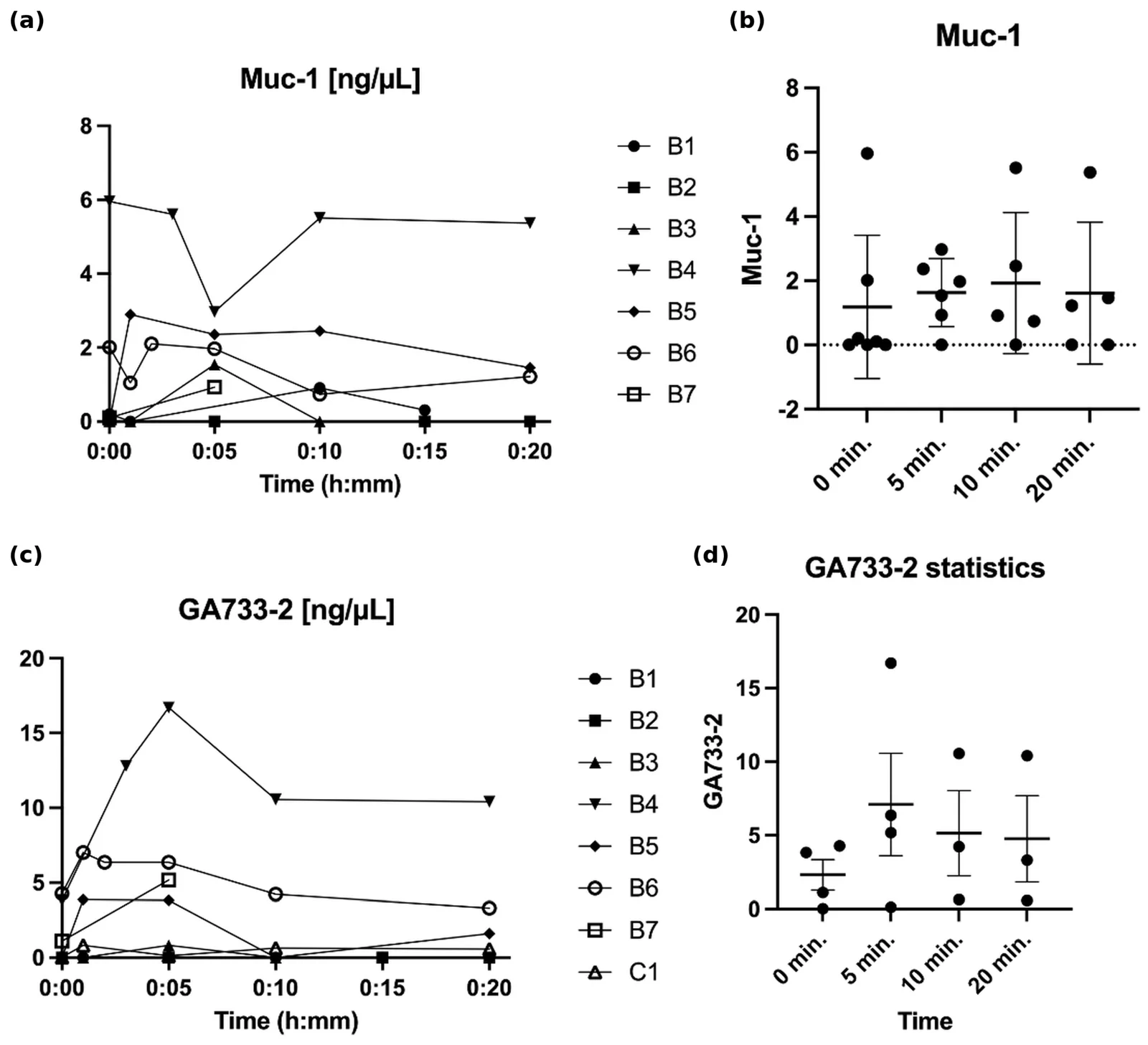
Changes in MUC-1 and GA733-2 levels in peripheral blood over time after kyphoplasty/vertebroplasty: (**a**) MUC-1 (ng/µL) in the individual breast cancer patients (B1–B7); (**b**) MUC-1 at 0, 5, 10, and 20 min in the breast cancer patients (individual values; horizontal line, mean; error bars, ± standard deviation; *n* = 7, 6, 5, and 5 observations at 0, 5, 10, and 20 min, respectively); (**c**) GA733-2 (ng/µL) in the individual breast cancer (B1–B7) and colon cancer (C1) patients; (**d**) GA733-2 at 0, 5, 10, and 20 min (individual values; horizontal line, mean; error bars, ± standard error of the mean; *n* = 4, 4, 3, and 3 observations, respectively). All individual observations are plotted; error bars extending below zero in (**b**) result from the symmetric representation of skewed, non-negative data with several values at or near zero and do not indicate negative concentrations.

Patients with GA733-2 marker positivity at baseline (*n* = 4; three with breast cancer and one with colon cancer) showed a 3.8-fold increase in mRNA expression at 5 min post-cement injection (paired t-test, nominal *p* = 0.026; 95% CI: 1.4–10.4). This exploratory finding rests on four paired observations and was not adjusted for multiple comparisons; the increase was not sustained at 10 or 20 min (Figure 1). Individual values of MUC-1 and GA733-2 for each patient and time point are shown in Figure 1a,c. In the exploratory correlation analyses, no association was found between marker levels and cement volume, vertebral location (thoracic vs. lumbar), or baseline marker positivity.

## 4. Discussion

An increase in circulating tumor cells (CTCs) in peripheral blood has been observed across various oncological surgeries, including those for intestinal, breast, and prostate tumors, suggesting that mechanical tumor manipulation during surgery may contribute to the release of tumor cells into circulation and increase metastatic risk. For example, in patients with non-small cell lung cancer, surgical intervention has significantly elevated CTC counts in pulmonary venous blood, indicating that tumor cell mobilization is influenced by surgical manipulation [15]. Similarly, in patients with localized breast cancer, a study found that peripheral CTC counts either remained stable or increased following tumor resection, indicating that surgery does not inherently decrease metastatic potential and may facilitate CTC release [16]. For certain tumors, CTC presence is associated with poorer survival and shorter recurrence-free intervals [17,18,19].

Our objective was to assess whether KP or VP for metastatic vertebral fractures is associated with the detection of, or an increase in, CTC-associated mRNA markers in peripheral blood. This investigation builds on the work by Mohme et al. [7], which analyzed fewer time points and relied solely on epithelial markers (CellSearch^®^ assay). Our findings obtained with the AdnaTest^®^ are in line with those of Mohme et al. By sampling frequently within the first 20 min after vertebral cement augmentation, we were able to describe the early perioperative dynamics of these markers. Marker detection was most consistent at 5 and 10 min after PMMA injection, when all nine biopsy-positive patients were marker-positive, whereas a quantitative increase was observed only for GA733-2 at 5 min, only in the small subgroup of four baseline-positive patients, and only at a nominal level of significance. These observations do not establish a generalized 5–10 min peak of CTC release across markers and tumor types. They do suggest, however, that a single sampling time point at 20 min, as used by Mohme et al., may not capture the earliest phase of the perioperative signal.

Moreover, our data indicate that mRNA-based marker detection is feasible in the perioperative setting of vertebral cement augmentation and may provide additional molecular information beyond EpCAM-based cell enumeration.

Recent years have seen the development of standardized methods for CTC quantification in peripheral blood, with the CellSearch^®^ assay (based on EpCAM epithelial markers) being the most widely used [20,21,22]. An mRNA-based approach, such as the AdnaTest^®^, may improve detection by capturing cells undergoing epithelial–mesenchymal transition (EMT), which are not typically detected by EpCAM-dependent methods. This approach allows for more detailed characterization of CTC subtypes. In clinical testing, the AdnaTest^®^ has shown sensitivity comparable to CellSearch^®^ when detecting two or more CTCs and complements CellSearch^®^ by increasing detection rates [22,23]. It should be noted, however, that the AdnaTest^®^ detects tumor-associated transcripts after immunomagnetic enrichment and neither enumerates intact cells nor assesses their viability; a positive result therefore indicates the presence of tumor-associated mRNA in the enriched cell fraction rather than the direct demonstration of viable CTCs.

With regard to the choice of markers for future studies, our data allow only a preliminary assessment. In the breast cancer patients, MUC-1 (6 of 7) and GA733-2 (5 of 7) were detected considerably more often than Her-2 (3 of 7), and GA733-2 was the only marker for which an early quantitative change was observed; in the single colon cancer patient, GA733-2 was likewise the only detectable marker. GA733-2 (EpCAM) and MUC-1 therefore appear to be the most promising candidates for a perioperative marker panel in breast cancer, whereas our data for prostate and colorectal cancer are too limited to recommend specific markers. Future studies should prespecify one primary marker and one primary time point, report individual-level positivity for every marker and time point, and complement the mRNA-based approach with a method that enumerates intact cells.

Whether the perioperative marker release observed in this study is of clinical relevance, and whether it can or should be reduced, cannot be answered with the present data. As hypotheses for future studies, two technical approaches appear worth investigating. One is the combination of KP/VP with pre-procedural radiofrequency ablation, which is intended to induce tumor cell death before cement injection and might thereby limit the release of viable cells [24,25]. The other might be vertebral body lavage before cement augmentation, which has been shown to reduce the systemic venous dissemination of bone marrow content in osteoporotic fractures [6,26]. We emphasize that neither approach has been shown to reduce perioperative tumor cell release or to improve oncological outcome after cement augmentation, and that our data do not demonstrate that reducing the observed molecular signal would translate into a clinical benefit. Although VP/KP use in metastatic cases has declined in our tertiary care center due to advances in radio-, chemo-, and immunotherapies, these procedures remain viable options for managing spinal instability and impending fractures in metastatic disease, particularly with pathological vertebral fractures associated with kyphotic deformity. KP offers the added benefit of realigning the spine, potentially improving load distribution, quality of life, and pain relief [27,28]. Given the extended survival in metastatic cases due to advancements in oncology, this approach warrants continued consideration. In the longer term, the development of bioactive, gradient-structured biomaterials for the spine, which is currently being explored for intervertebral disc regeneration [29], may eventually offer alternatives to PMMA-based augmentation, although such approaches remain far from clinical application in metastatic disease.

### Limitations

Although this study was prospective, enrollment was limited to 11 patients over a practical timeframe, which may limit the robustness of our findings. However, the frequent sampling at short intervals (1, 5, 10, and 20 min) allowed us to observe the early dynamics of CTC-associated markers during VP and KP procedures. One limitation was the absence of longer-term analyses (e.g., at 2 h or days post-procedure) to track CTC elimination, as Mohme et al. previously addressed this timeframe [7]. Although we observed a transient increase of one CTC-associated marker in a small subgroup of patients, we cannot assess its clinical relevance. To make statements about overall prognosis, a substantially larger number of patients and longer clinical follow-ups would be needed. Furthermore, it remains to be evaluated to what extent additional chemotherapy or radiotherapy might affect the viability of CTCs in the venous circulation. It is also unknown whether the dissemination of tumor cells from the metastasis through VP/KP could lead to further metastases. For this, larger clinical studies would also be necessary.

Several further limitations should be acknowledged. First, the cohort was heterogeneous with respect to the primary tumor, with a predominance of breast cancer (7 of 10 evaluable patients); the prostate (*n* = 1 evaluable) and colorectal (*n* = 1) subgroups were too small for any separate analysis, so that our results are essentially driven by the breast cancer patients. Second, the tumor-specific marker panels differed between tumor types, which limits the comparability of detection rates across primary tumors. Third, blood was drawn via a central venous catheter in six and via a peripheral venous catheter in five patients; although the route remained constant within each patient, central and peripheral sampling may differ in the concentration of tumor-derived material, and the small sample size did not allow adjustment for this heterogeneity. Fourth, the study lacked a control group without cement augmentation (e.g., vertebral access and biopsy alone or a sham procedure), so that the contribution of the cement injection itself to the observed marker dynamics cannot be separated from that of vertebral access and biopsy. Fifth, the single biopsy-negative patient cannot serve as a true disease-negative control, since a negative vertebral biopsy in a patient with imaging-based evidence of metastatic disease may reflect a sampling limitation. Sixth, the assay detects tumor-associated mRNA after immunomagnetic enrichment; it neither enumerates intact cells nor determines their viability, so that it remains unknown whether the detected molecular signals represent viable, metastasis-competent tumor cells. The statistical analysis was exploratory: no primary marker or time point was prespecified, several markers and time points were compared without adjustment for multiplicity, and the only nominally significant result rests on four paired observations, for which neither the normality assumption nor a non-parametric confirmation can be meaningfully assessed and which would not withstand correction for multiple testing. In addition, the analytical reproducibility of the assay was not formally assessed, and laboratory personnel were not formally blinded. Our results should therefore be regarded as exploratory and hypothesis-generating.

## 5. Conclusions

In this prospective pilot study, CTC-associated, tumor-specific mRNA markers were detectable in the peripheral blood of all patients with histologically confirmed vertebral metastases within the first 20 min after cement augmentation, most consistently at 5 and 10 min, and a transient increase in GA733-2 was observed at 5 min in a small subgroup of baseline-positive patients. These findings are compatible with a procedure-related release (intravasation) of tumor-derived material into the circulation, but they do not demonstrate the dissemination of intact, viable tumor cells, and their prognostic relevance remains unknown. The mRNA-based approach proved feasible in the perioperative setting, and sampling within the first 10 min after cement injection appears to be the most informative window for future studies. Larger, adequately powered studies with prespecified primary markers and time points, individual-level reporting, a control group, and longer follow-up are required before perioperative strategies to limit tumor cell release—such as pre-procedural radiofrequency ablation or vertebral body lavage—can be evaluated.

## Figures and Tables

**Table 1:** Patient demographics and procedural characteristics.

Patient	Primary Tumor	Age (Years)	Weight (kg)	Height (m)	BMI (kg/m^2^)	Sex	Extraspinal Metastases	SINS	Vertebral Level	Cement Volume (mL)
B1	Breast	63	69	1.60	27.0	f	Y	14	L3 + L5	8
B2	Breast	58	59	1.62	22.5	f	Y	12	T12	5.5
B3	Breast	74	55.2	1.62	21.0	f	Y	12	T12	4.5
B4	Breast	75	100	1.64	37.2	f	Y	12	L4	10
B5	Breast	39	58	1.67	20.8	f	N	15	L4 + L5	4.4
B6	Breast	70	59	1.67	21.2	f	Y	13	L4	8
B7	Breast	70	62	1.67	22.2	f	Y	9	L1	6.5
P1	Prostate	66	82	1.81	25.0	m	Y	14	L4	10
P2	Prostate	67	86	1.84	25.4	m	Y	8	T12	6
P3	Prostate	77	85	1.74	28.1	m	Y	11	L4	7
C1	Colon	66	52	1.65	19.1	f	Y	14	T7	4.5

BMI, body mass index; SINS, Spinal Instability Neoplastic Score; f, female; m, male; Y, yes; N, no.

## Data Availability

The data presented in this study are available on request from the corresponding author due to privacy and ethical restrictions.

## Abbreviations

The following abbreviations are used in this manuscript:

VP	vertebroplasty
KP	(balloon) kyphoplasty
PMMA	polymethyl methacrylate
CTC	circulating tumor cell
EpCAM	epithelial cell adhesion molecule
EMT	epithelial–mesenchymal transition
SINS	Spinal Instability Neoplastic Score
BMI	body mass index
PCR	polymerase chain reaction
cDNA	complementary DNA
CI	confidence interval
SD	standard deviation
